# Ru and Se Co-Doped Cobalt Hydroxide Electrocatalyst for Efficient Hydrogen Evolution Reactions

**DOI:** 10.3390/molecules28155736

**Published:** 2023-07-28

**Authors:** Weizhong Peng, Yuting Yuan, Chao Huang, Yulong Wu, Zhaohui Xiao, Guanghui Zhan

**Affiliations:** State Key Laboratory of Marine Resource Utilization in South China Sea, School of Materials Science and Engineering, Hainan University, Haikou 570228, China; 20080500210022@hainanu.edu.cn (W.P.); 20080500210040@hainanu.edu.cn (Y.Y.); 21210805000018@hainanu.edu.cn (C.H.); 20203100020@hainanu.edu.cn (Y.W.); xiaozh@hnu.edu.cn (Z.X.)

**Keywords:** electrodeposition, material compounding, elemental doping, electrolytic water, HER

## Abstract

The development of efficient electrocatalysts for hydrogen evolution reactions is an extremely important area for the development of green and clean energy. In this work, a precursor material was successfully prepared via electrodeposition of two doping elements to construct a co-doped cobalt hydroxide electrocatalyst (Ru-Co(OH)_2_-Se). This approach was demonstrated to be an effective way to improve the performance of the hydrogen evolution reaction (HER). The experimental results show that the material exhibited a smaller impedance value and a larger electrochemically active surface area. In the HER process, the overpotential was only 109 mV at a current density of 10 mA/cm^2^. In addition, the doping of selenium and ruthenium effectively prevented the corrosion of the catalysts, with the (Ru-Co(OH)_2_-Se) material showing no significant reduction in the catalytic performance after 50 h. This synergistic approach through elemental co-doping demonstrated good results in the HER process.

## 1. Introduction

Considering the current energy crisis and serious environmental pollution levels, the search for green energy sources as an alternative to traditional energy sources has become an urgent concern [1,2,3]. Hydrogen energy is currently one of the most important green energy sources, and the HER is a key step in the process of hydrogen electrolysis, which is a reduction reaction to reduce the activation energy of the reaction and accelerate the combination of hydrogen ions and electrons through catalysts to achieve efficient hydrogen production [4,5,6]. In recent years, with the increasing demand for green energy, research on HER catalysts has received significant attention [7]. Researchers have improved the performance of catalysts by controlling their structure, composition, and surface activity sites to achieve more efficient HERs [8,9,10]. At present, many efficient HER catalysts have been discovered, such as platinum group metals, transition metal compounds, and carbon-based materials [11,12,13,14,15]. However, these catalysts still have drawbacks, such as their high cost and scarcity; therefore, further research is required to develop more efficient and low-cost HER catalysts for the sustainable development of hydrogen energy [16,17,18]. In general, the electrochemical hydrolysis reaction consists of two key half-reactions, namely, the hydrogen evolution reaction at the cathode (HER) and the oxygen evolution reaction at the anode (OER) [17].

Doping is one of the most common methods for improving the HER activity of catalysts [19]. Doping with noble metals (e.g., Pt and Ir) is widely employed in HER catalysts due to the metals’ excellent electrocatalytic properties [20,21,22,23]. However, the high cost of noble metal doping limits its application scope. In contrast, doping with non-metal anions (e.g., anions of N, P, S, and Se) has the advantages of low cost, abundant resources, and environmental friendliness, and has gained widespread attention in the HER field [24,25,26,27]. Non-metallic doping can improve the HER activity via mechanisms such as modulating the electronic structure of catalysts and increasing the reactivity of active sites, which has become one of the hot spots of this research area [28,29]. The utility of noble metal doping is due to the metal’s higher electron density and the hydrogen bonds formed between metal atoms and water molecules on the catalyst surface, increasing the HER rate [30,31]. Sharma et al. reported a computational study of Zn metal atom doping on the surface of one-dimensional NiMoO_4_ nanorods, which improved the redox chemistry. The results of this study are attributed to the enhancement of the charge transfer between the surface atoms and electrolyte ions [32,33]. Non-metal anion doping, on the other hand, increases the activity of the HER by changing the electronic structure and active sites of the catalyst through the introduction of dopant atoms [19,34]. Among them, dopant atoms such as N and P can increase the density and number of active sites on the catalyst surface, while dopant atoms such as S and Se can modify the charge distribution on the catalyst surface and, thus, improve its HER activity. However, the efficiency of non-metal doping is far from satisfactory. In contrast, noble metal electrolytic water reactions are highly active, highlighting the potential of co-doping noble metals and non-metals with transition metal compounds [35,36]. Ruthenium is a cost-effective noble metal that has been shown to improve electrochemical reaction efficiencies by ruthenium loading [37,38]. Therefore, the co-modification of transition metal compounds by ruthenium loading alongside the doping of non-metallic elements is a promising method for improving catalytic performance. Chen et al. synthesized 3D flower-like Ru-doped bimetallic phosphide (Ru-NiCoP/NF) electrocatalysts; supported Ru-doping in the Ni site may enhance the catalytic performance of NiCoP by providing a more active bifunctional site for OER and HER by DFT theoretical calculations, and the HER overpotential at 10 mA/cm^2^ was 44 mV under alkaline electrolyte conditions [39]. Liu et al. prepared a core–shell structured electrocatalyst for HERs under alkaline conditions with a selenium-doped phosphide layer on the inside and a nitrogen-doped carbon layer on the outside, which achieved a solar hydrogen production efficiency of 10.2%, with long-term stability at high current densities [40]. Qiu et al. revealed the dynamic evolution of the local geometry and electronic structure of nickel–iron carbonate hydroxide hydrate (NFCH) electrodes under oxidation potential. The results show that, in the early stage of cyclic voltammetry (CV) cycles, the irreversible redox of Ni cations will lead to the phase transition of the porous NF-CH-O nanosheets (nickel ferrite (NFO) intercalated nickel–iron carbonate hydroxide hydrate), thus forming an intact NiFe layered double hydroxide (NF-LDH-O) nanosheet with high surface roughness, which has excellent OER catalytic activity and stability [41]. Qiu et al. designed and prepared an MgCo_2_O_4_@WO_3_ core–shell heterostructure (MCW CSHS) that is constructed by the simple one-step hydrothermal coordination of the spinel oxide MgCo_2_O_4_ (as the electron acceptor) and the WO_3_ semiconductor (as the electron donor). Under solar illumination, the electrocatalytic activity of MCW CSHS is significantly improved due to the electronic structure regulation, yielding 50 mA·cm^−2^ at overpotentials of 243 mV for oxygen evolution reactions and 161 mV for hydrogen evolution reactions in 1 M KOH [42]. Qiu et al. prepared Nb/Fe co-doped Ni-Ses (NbFe-Ni_x_Se_y_) electrocatalyst that exhibits the prominent oxygen and hydrogen evolution reaction (OER/HER) properties, with low overpotentials of 237 and 226 mV at 50 mA·cm^−2^, respectively. The alkaline water electrolyzer with NbFe-Ni_x_Se_y_ as both anodic and cathodic electrodes only requires a cell potential of 1.7 V to reach 50 mA cm^−2^ in a continuous operation of 50 h [43].

Therefore, we attempted to add Ru and Se to cobalt hydroxide to improve its electrochemical activity. A three-step method was devised for the preparation of (Ru-Co(OH)_2_-Se) layered sheet-like structures. The precursors were grown directly onto the Ti network via electrodeposition, then immersed in RuCl_3_ solution containing Ru^3+^ to load Ru on the catalyst surface, and, finally, Se doping was performed. Due to the doping of Ru and Se, Ru-Co(OH)_2_-Se has a stable structure and good electrochemical activity, and the overpotential is significantly reduced in the HER. Co(OH)_2_ was prepared by a simple electrodeposition method and subsequently modified by co-doping with cations and anions. Through the synergistic effect of anions and cations, Ru-Co(OH)_2_-Se has good HER performance, with a significant reduction in overpotential, and can work for a long time without degradation. This is useful for optimizing the catalyst approach.

## 2. Results and Discussion

### 2.1. Structural and Morphological Characterization

**Figure 1:**
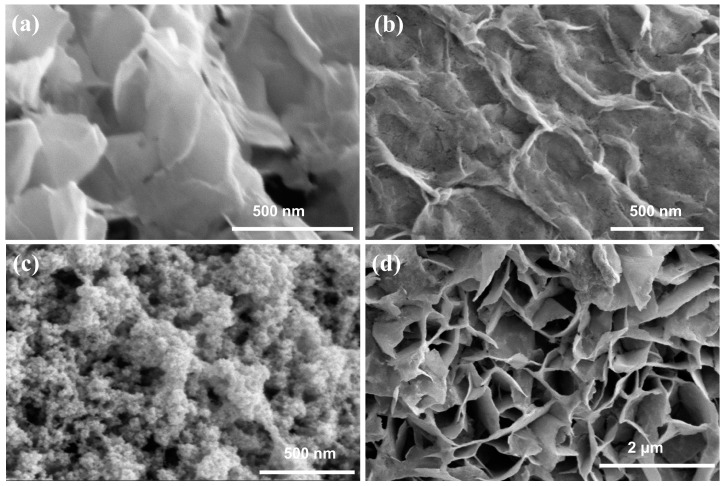
SEM images of (**a**) Co(OH)_2_, (**b**) Co(OH)_2_-Se, (**c**) Ru-Co(OH)_2_, and (**d**) Ru-Co(OH)_2_-Se.

Field emission scanning electron microscopy (FESEM) was used to characterize the microscopic morphology. As shown in Figure 1a, Co(OH)_2_ has a porous lamellar structure. Co(OH)_2_-Se (after selenium doping) exhibited a significant reduction in pores, and even a partial closure of pores, on the basis of the porous lamellar structure of the Co(OH)_2_ substrate, but this did not cause a fundamental change in its structure, as shown in Figure 1b. As shown in Figure 1c, Ru-Co(OH)_2_ formed closely stacked clusters on the substrate material, which were uniformly distributed on the substrate surface. The SEM image of Ru-Co(OH)_2_-Se is shown in Figure 1d. The co-doping of Ru and Se did not affect the structure of the sample significantly, and the structure after co-doping remained porous and lamellar. As shown in Figure S2, the structure of Ru-Co(OH)_2_-Se was changed, with the appearance of hexagonal cubes when post-HER. In the elemental EDS distribution diagram of Ru-Co(OH)_2_-Se, the elemental content of Co as the base material shown in Figure S1 is the highest among Co, Se, and Ru. As shown in Figure S1c,d, both Se and Ru are loaded onto the surface of the material, proving the successful co-doping of the sample. As shown in Figure S3c,d, both Se and Ru for Ru-Co(OH)_2_-Se are still loaded onto the surface of the material when post-HER, proving elemental did not shed after the HER reaction.

**Figure 2:**
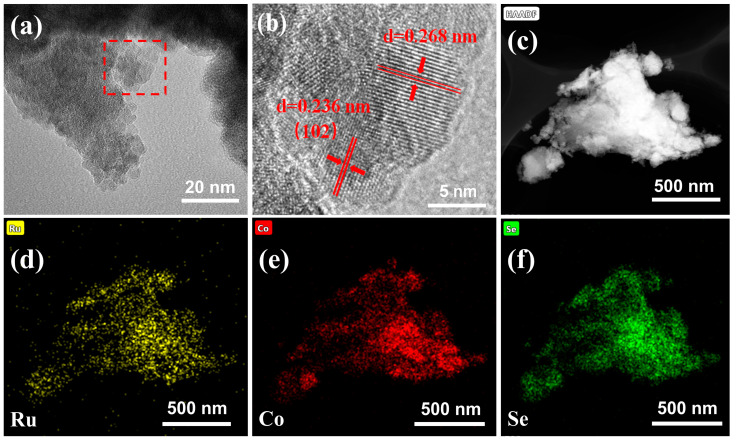
TEM images of (**a**,**b**) Ru-Co(OH)_2_-Se. HAADF-STEM (High-Angle Annular Dark Field-Scanning Transmission Electron Microscopy) images of (**c**) Ru-Co(OH)_2_-Se. EDS elemental mapping images of (**d**) Ru, (**e**) Co, and (**f**) Se.

Transmission electron microscopy (TEM) was used to characterize the morphology of the co-doped electrode. The microscopic morphology of Ru-Co(OH)_2_-Se can be observed in Figure 2a as a lamellar structure formed by multiple closely connected lamellar layers. Furthermore, in the high-resolution 5 nm TEM image shown in Figure 2b, clearer lattice stripes of the material can be observed. The spacing of the lattice stripes was subsequently measured and determined to be 0.236 nm, which is similar to that of the crystal plane. The crystal plane spacing matches the (102) crystal plane of Co(OH)_2_ (JCPDS No.51-1731) [44]. The elemental distribution of the Ru-Co(OH)_2_-Se lamellar structure was examined via energy-dispersive X-ray spectroscopy (EDS). As shown in Figure 2c, Ru, Co, and Se elements are distributed in the Ru-Co(OH)_2_-Se layer sheet structure after co-doping the electrode, which again proves the successful co-doping of the sample.

X-ray diffraction (XRD) was performed to identify and analyze the physical phase and crystal structure of the samples prepared on titanium mesh. As shown in Figure S4, three distinct diffraction peaks appear at 2θ = 38.52°, 40.20°, and 70.58°, which are associated with the titanium (Ti) in the titanium mesh substrate [45]. In addition, there is a very small, broad peak, and this peak is not related to the substrate titanium mesh and, thus, may be an error during the analysis. For the catalyst electrode sample material, X-ray diffraction was performed, and the test range was 5–85°. In Figure S4, the diffraction peak at 2θ = 9.72° corresponds to the (001) crystal plane, the diffraction peak at 23.2° corresponds to the (012) crystal plane, and the diffraction peak at 51.66° corresponds to the (110) crystal plane of Co(OH)_2_ (JCPDS No. 51-1731) [46]. The XRD morphological and structural characterization conducted above further demonstrates that the material on the surface of the titanium mesh is mainly the base material Co(OH)_2_.

In order to investigate the detailed surface chemical environment of the catalyst, X-ray photoelectron spectroscopy (XPS) was used. XPS spectroscopy was also performed on Ru-Co(OH)_2_-Se after the HER to analyze the elemental valence transitions on the surface of the catalyst material before and after the HER. The spectra in Figure S5 show the coexistence of Ru, Co, and Se in Ru-Co(OH)_2_-Se. The Ru 3p spectrum can be divided into two fitted peaks, indicating the existence of Ru elements in Ru-Co(OH)_2_-Se, and Ru was not shed before and after the HER. The fitted peaks in Figure S5 match perfectly with Ru^0^ 3p_3/2_ (461.8 eV) and Ru^0^ 3p_1/2_ (483.8 eV) [47], indicating that the Ru in Ru-Co(OH)_2_-Se exists in a metallic state, which may be related to its reduction during the annealing process. The peaks of Se shift to high energy before and after the HER, indicating that Se loses electrons during the HER process. As shown in Figure S5, compared with the pristine Co(OH)_2_, the Co 2p_3/2_ peak shifted to the low-binding-energy direction, and the Co valence state increased after Ru doping, indicating that Ru was loaded on the catalyst surface in the form of a redox replacement reaction during Ru doping. In contrast, after the Se treatment, the Co 2p_3/2_ shifts toward the high-binding-energy direction, indicating that the valence state of Co decreases. And after simultaneous Ru doping and Se doping treatment, Co 2p_3/2_ is further positively shifted, which also means that the electron distribution environment around the Co ion is transformed into an electron-rich state, which is favorable for the attraction of H_2_O to the Co atoms on the catalyst surface and its conversion to H_2_, thus reducing the dissociation energy of water [48]. The O 1s spectrum of pristine Co(OH)_2_ can be divided into two fitted peaks attributed to Co-OH (O_2_) and surface adsorbed oxygen (O_1_), respectively. Further after doping with Ru, a new fitted peak can be divided into a new peak attributed to O_3_ (Ru-O), surface Ru successfully loaded on the surface of Co(OH)_2_ [49].

Subsequently, Raman spectroscopy was performed on the co-doped and single-doped materials separately to determine the molecular structure [50]. As shown in Figure S6, there is a strong Raman peak in the Raman spectrum of Ru-Co(OH)_2_, and the position of this peak is 517.8 cm^−1^, which corresponds to the stretching vibration of the Co-O bond [50]. The peaks in the Raman spectra of Co(OH)_2_-Se are located at 522 cm^−1^ and 685.5 cm^−1^, and there is a slight shift in the peaks in the Raman spectra of Ru-Co(OH)_2_-Se upon co-doping, in which the Raman peaks are located at about 525.3 cm^−1^ and 692.7 cm^−1^, and Raman features appear near 690 cm^−1^. The peak near 690 cm^−1^ may be caused by the interactions between the doped Se and the original material due to selenization.

### 2.2. Electrochemical Performance

**Figure 3:**
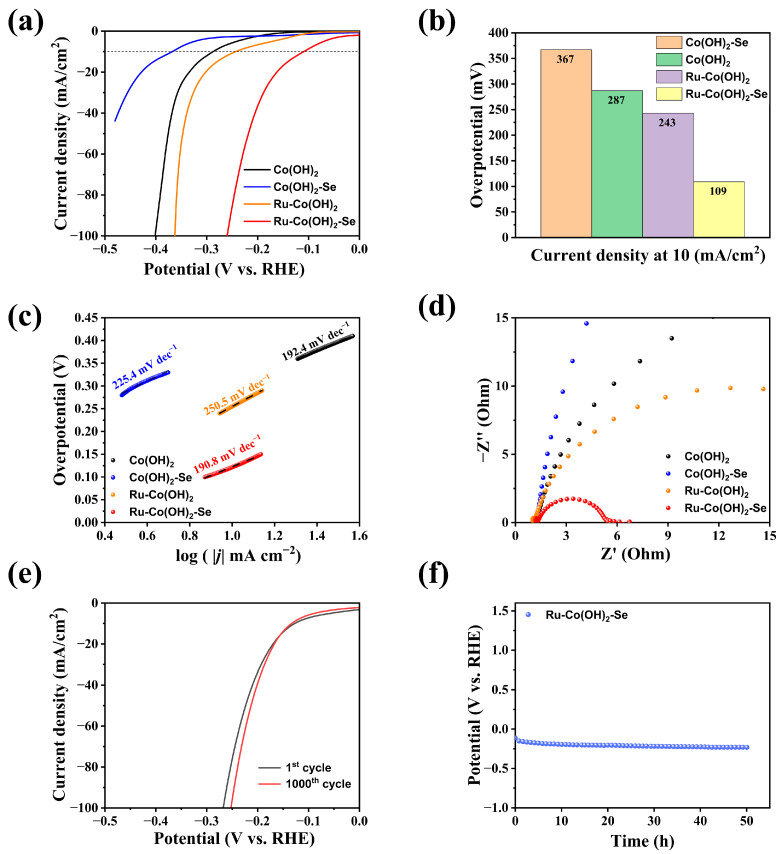
(**a**) LSV polarization curves. (**b**) Overpotential at 10 mA/cm^2^. (**c**) Tafel curves. (**d**) Nyquist plot. (**e**) LSV polarization curve before and after 1000 CV cycles. (**f**) Timing of the potential curve.

The electrocatalytic HER performance of the four electrode materials was tested in an alkaline solution (1 M KOH). The electrocatalytic HER performance was tested by doping the base Co(OH)_2_ material and the other three prepared samples. In Figure 3a, the electrode samples co-doped with Ru and Se under different doping elements showed the best catalytic performance compared with other electrodes. An accurate comparison of the HER performance of Co(OH)_2_, Ru-Co(OH)_2_, Co(OH)_2_-Se, and Ru-Co(OH)_2_-Se was conducted, and after the analysis of Figure 3a, it can be concluded that Ru-Co(OH)_2_-Se obtains a current density of 10 mA/cm^2^ at an overpotential of 109 mV. The overpotentials of Co(OH)_2_, Ru-Co(OH)_2_, and Co(OH)_2_-Se were 287 mV, 243 mV, and 367 mV, respectively, at a current density of 10 mA/cm^2^. The overpotential at 10 mA/cm^2^ is an important performance evaluation index, as shown in Figure 3b, and indicates that the co-doped Ru-Co(OH)_2_-Se has a much better catalytic performance than Co(OH)_2_-Se. The catalytic performance of the co-doped Ru-Co(OH)_2_-Se is much better than that of Co(OH)_2_, Ru-Co(OH)_2_, and Co(OH)_2_-Se. Figure 3c shows the Tafel curves of the above four electrodes, where the Tafel slope of Ru-Co(OH)_2_-Se is 190.8 mV/dec, which is smaller than that of Co(OH)_2_ (192.4 mV/dec), Ru-Co(OH)_2_ (250.5 mV/dec), and Co(OH)_2_-Se (225.4 mV/dec). The Tafel curve slope can depict the HER rate with the change in potential; the smaller the slope, the smaller the potential changes that can affect the HER rate, while the larger the slope, the larger the potential changes that can affect the HER rate. The Tafel plots also demonstrate that Ru-Co(OH)_2_-Se has a good electrocatalytic performance compared to the other three electrodes.

Electrochemical impedance spectroscopy (EIS) was performed at an overpotential of 109 mV to compare the charge transfer rates of the electrodes and determine the charge transfer efficiency. The impedance value of each electrode can be estimated from the diameter of the semicircle shown in the test graph, and the smaller the impedance value, the faster the charge transfer efficiency. As shown in Figure 3d, Ru-Co(OH)_2_-Se has the smallest electrochemical impedance (4.2 Ω), and this value is much smaller than the values of Ru-Co(OH)_2_ (24.9 Ω), Co(OH)_2_ (42.8 Ω), and Co(OH)_2_-Se (218.6 Ω). This indicates that the charge transfer rate of Ru-Co(OH)_2_-Se is significantly enhanced after co-doping, and the HER kinetics are accelerated.

As shown in Figure S7a–c, the sweep rates of the cyclic voltammetry (CV) tests were 10, 20, 30, 40, and 50 mV/s, and the values of the differences in the current densities (Δj) at the same potential were greater for Ru-Co(OH)_2_-Se than for Ru-Co(OH)_2_ and Co(OH)_2_-Se. As shown in Figure S7d, the values of the differences in the current densities (Δj) by sweep rate and the slopes (C_dl_) fitted to the data of Ru-Co(OH)_2_-Se, Ru-Co(OH)_2_, and Co(OH)_2_-Se were 0.3, 0.25, and 0.14 mF/cm^2^, respectively. The number of active sites can be judged by the magnitude of the electrochemically active surface area. ECSA values of 7.5 cm^2^ for Ru-Co(OH)_2_-Se, 6.25 cm^2^ for Ru-Co(OH)_2_, and 3.5 cm^2^ for Co(OH)_2_-Se can be derived from the equation ECSA = (C_dl/_C_s_) × ASA. It is easy to observe from the magnitude of the ECSA values that the synergistic effect of co-doping increases the electrochemically active surface area of Ru-Co(OH)_2_-Se and also increases the number of active sites. Thus, the combination of a smaller electrochemical impedance and a larger electrochemical active area leads to a significantly better HER catalytic performance for the co-doped Ru-Co(OH)_2_-Se compared to the single-doped Ru-Co(OH)_2_ and Co(OH)_2_-Se.

The stability of the co-doped material, Ru-Co(OH)_2_-Se, in 1 M KOH electrolyte was tested by using it as a working electrode, alongside graphite as the counter electrode and a saturated calomel electrode (SCE, 0.241 V) as the reference electrode in the potential range of −0.533–0.167 V vs. the LSV curves, which were measured once after the CV cycles and compared with the initial LSV curves before 1000 CV cycles. Shown in Figure 3e are the LSV curves before and after the test overlap, and the electrocatalytic performance is improved, which may be due to the activation effect of 1000 CV cycles and indicates that the electrode material has better cycling stability in the 1 M KOH electrode solution. After performing co-doping, the Ru-Co(OH)_2_-Se electrode has good catalytic stability, and the reaction of Ru-Co(OH)_2_-Se occurs at a current density of 100 mA/cm^2^ for 50 h. As shown in Figure 3f, the potential remains essentially constant over 50 h. Meanwhile, as shown in Figure S8, the Ru-Co(OH)_2_-Se electrode has good catalytic stability in 30 wt% KOH for 50 h.

### 2.3. Reaction Mechanism

**Figure 4:**
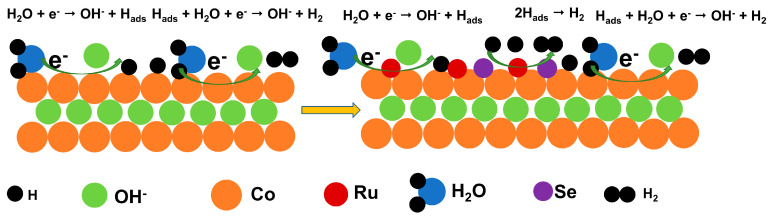
Schematic diagram of HER mechanism.

Figure 4 briefly illustrates HER mechanism. Cobalt is a transition metal, with abundant reserves and low cost, and has a wide range of applications in electrocatalysis. However, due to the weak bond energy of the Co-H bond, the adsorption ability of Co sites is weak, and the HER activity is not high. Ru is a low-cost noble metal compared to Pt, and the bond energy of Ru-H bond is closer to that of the Pt-H bond, and this strong interaction is beneficial to the adsorption of H_2_O, but it will reduce the resolution efficiency of H_2_. Therefore, the introduction of Ru into Co can balance the metal–H interaction. The Ru site promotes the adsorption of H_2_O molecules, the Co site promotes the resolution of H_2_, and a balance is reached between the catalyst activity and adsorption strength. The addition of Se increases the layer spacing (the atomic size of Se is larger than that of OH^−^) and changes the edge electronic structure, which further improves the HER performance of electrolytic water, responding to the synergistic effect of Ru and Se doping and exhibiting more excellent catalytic performance. The steps of HER primitives under alkaline conditions are as follows: H_2_O + e^−^ → OH^−^ + H_ads_ (Volmer) (1), H_ads_ + H_2_O + e^−^ → OH^−^ + H_2_ (Heyrovsky) (2), or 2H_ads_ → H_2_ (Tafel) (3). The “ads” represents adsorbed state of intermediates (Hads). The first step of the HER is to generate H^+^ by reaction with H_2_O in 1 M KOH. The second step depends on the free energy of hydrogen adsorption (∆G_H_^+^) at the active site; the Ru active site Tafel reaction adsorbs H^+^ and H^+^ to generate H_2_, while the Co active site reacts with H_2_O and H^+^ to generate H_2_. The reason for different reaction paths is that the ∆G_H_^+^ on Ru metal is very small, while the ∆G_H_^+^ on Co metal is very high [51]. Se changes the edge electronic structure, which further improves the HER performance of electrolytic water.

## 3. Materials and Methods

### 3.1. Preparation and Characterization

**Figure 5:**
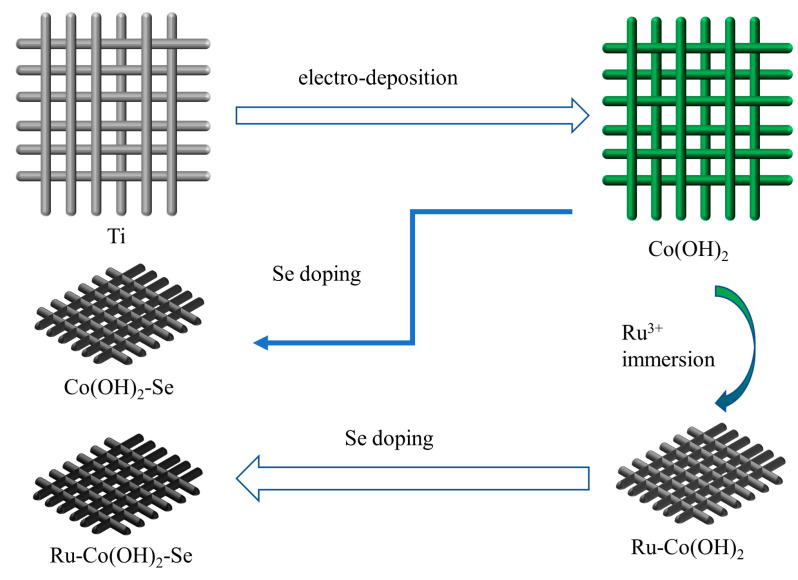
Schematic diagram of the electrode material preparation.

Figure 5 briefly illustrates the synthetic process for ruthenium and selenium co-doped materials. Regarding the preparation method for the catalyst electrode, the steps are as follows: The titanium mesh was cut using a paper cutter at a size of 2 cm × 3 cm, and the cut titanium mesh pieces were ultrasonically cleaned in ethanol and deionized water for 15 min each and air dried after cleaning. The titanium mesh was placed in a 50 mM solution of cobalt nitrate hexahydrate. The above solution was used as the electrolyte, with graphite as the counter electrode; a saturated calomel electrode (SCE, 0.241 V) as the reference electrode; and titanium mesh clamped to the electrode clamp as the working electrode. The material was prepared via electrodeposition at a constant potential of −1.0 V vs. SCE for 600 s. To grow Co(OH)_2_ on the titanium mesh, an electrochemical workstation was used. After drying in air, the prepared samples were placed in a 10 mM solution of ruthenium chloride and fully immersed for 30 min in the dark in order to allow sufficient loading of Ru^3+^ onto the samples. These samples were denoted as Ru-Co(OH)_2_. The Co(OH)_2_ and Ru-Co(OH)_2_ samples were placed in a porcelain boat and put into a quartz tube, along with 0.5 g of selenium powder. Then, selenium doping was performed under a nitrogen atmosphere at 350 °C for 2 h in a tube furnace. The doped samples, Ru-Co(OH)_2_-Se and Co(OH)_2_-Se, were successfully obtained from Ru-Co(OH)_2_ and Co(OH)_2_, respectively.

The microscopic morphology of the samples was characterized by field emission scanning electron microscopy (SEM, Verios G4 UC, Thermo Fisher Scientific, Waltham, MA, USA) and field emission transmission electron microscopy (TEM, Talos F200X G2, Thermo Fisher Scientific, Waltham, MA, USA). X-ray diffraction (XRD, DX-2700BH, HAOYUAN, Dandong, China) was also used to characterize the structure of the samples, with a test start angle of 5° and an end angle of 85°. The surface elemental chemical environment of the samples and the valence information of the elements were determined via X-ray photoelectron spectroscopy (XPS, ESCALAB 250Xi, Thermo Fisher Scientific, Waltham, MA, USA) with the following specification parameters: X-ray source, Al Ka; energy, 1486.6 eV; and a binding energy, determined by the carbon (C) standard peak (C 1s) of 284.8 eV for correction. The molecular structure of the samples was also analyzed via Raman spectroscopy (Reflex in Via, Renishaw plc, Gloucestershire, UK). The excitation wavelength of the laser source was 514 nm, and the test range was 100–1100 cm^−1^.

### 3.2. Performance Testing

The catalysts were tested for electrocatalytic hydrogen generation in a 1 M KOH solution using the four electrodes prepared above as working electrodes, graphite as the counter electrode, and a saturated calomel electrode (SCE, 0.241 V) as the reference electrode, combined in a three-electrode system. The measured electrode potential was converted to reversible hydrogen electrode (RHE) potential using the formula E_RHE_ = E + 0.059 × pH + E_SCE_. The electrolyte is a potassium hydroxide solution; thus, the pH theoretical value is taken as 14 and the pH Measured value is taken as 13.77, and as a saturated calomel electrode is the reference electrode, the E_SCE_ is 0.241 V. First, the electrode material underwent linear scanning voltammetry (LSV) in the potential range of −0.533–0.167 V (vs. RHE) and at a sweep rate of 5 mV/s. In the test, the used area of the catalyst material was 1 cm^2^, and I-R compensation was applied to the test results based on the internal resistance of the system measured via an impedance test. The obtained Tafel slope was also fitted to the LSV curve after I-R compensation.

Electrochemical impedance spectroscopy (EIS) was performed in a frequency range of 100–0.01 Hz with an effective electrode area of 1 cm^2^, and all the samples were tested at a potential of 1 M KOH −1.2 V (vs. SCE) with a step potential of 10 mV.

Cyclic voltammetry (CV) was performed at different sweep speeds of 10, 20, 30, 40, and 50 mV/s, with an effective area of 1 cm^2^ and a potential range of 0.917–1.017 V (vs. the RHE), to determine the relationship between the change in the current density and the sweep speed. The electrochemically active surface area (ECSA) is also an important performance indicator for a working electrode and can be calculated from the slope of the straight line obtained via CV and fitting the change in the current density with the sweep speed, where the value of the double-layer capacitance is C_dl_ = Δj/2ν, where ν is the sweep rate and Δj is the difference in current density at the same sweep rate and at the same potential. Subsequently, the electrochemically active surface area (ECSA) of the electrode material can be deduced from the equation ECSA = (C_dl_/C_s_) × ASA. C_dl_ is the electrochemical bilayer capacitance, C_s_ is the specific capacitance, and ASA is expressed as the area of the electrode involved in the reaction.

The electrode stability test consisted of the following two parts: First, LSV was performed on the catalyst material before and after 1000 CV cycles and a potential range of −0.533 to 0.167 V (vs. the RHE), and at a sweep rate of 5 mV/s, in a 1 M KOH solution. Next, the LSV test curves obtained before and after 1000 CV cycles were compared to determine the stability, where the sweep rate of the LSV test was 5 mV/s. The electrolyte has not been changed after CV before LSV. Second, the E-T (Electrode potential-Time) test was performed for 50 h at a constant current density of 100 mA/cm^2^ to examine the change in potential and to determine the stability of the catalyst material.

## 4. Conclusions

The electrochemical properties of the studied catalysts were improved by performing elemental co-doping. Ruthenium and selenium co-doping optimized the water and intermediate content in the active sites, and the synergistic effect of the metal and non-metal resulted in a significant reduction in the interfacial charge transfer rejection. This was due to the stronger electronegativity of Ru enhancing the electron transfer ability, and its higher electron density and the hydrogen bonding between metal atoms and water molecules on the catalyst surface increasing the HER rate. Se doping was performed through the introduction of dopant atoms to change the electronic structure and active sites of the catalyst, thus improving the activity of the HER. The catalyst’s performance was favorable. In summary, by co-doping the material with two elements, we successfully synthesized an electrocatalyst (Ru-Co(OH)_2_-Se) with a stable sheet structure and good performance. As expected, the Ru-Co(OH)_2_-Se exhibited a favorable HER performance (η_10_ = 109 mV) in an alkaline electrolyte (KOH), showing a substantial improvement compared to the pre-doped and single-doped materials. This study provides a method for further enhancement and modification of hydroxide catalysts.

## Data Availability

No data were used for the research described in the article.

## Supplementary Materials

The following supporting information can be downloaded at: https://www.mdpi.com/article/10.3390/molecules28155736/s1, Figure S1: (a) SEM image of Ru-Co(OH)_2_-Se. EDS elemental mapping images of (b) Co, (c) Se, (d) Ru.; Figure S2: SEM image of post-HER Ru-Co(OH)_2_-Se.; Figure S3: (a) SEM image of post-HER Ru-Co(OH)_2_-Se. EDS elemental mapping images of (b) Co, (c) Se, (d) Ru.; Figure S4: X-ray diffraction (XRD) patterns of each electrode material.; Figure S5: (a) XPS spectra of the electrode material. (b) O 1s spectrum, (c) Co 2p spectrum, (d) Se 3d spectrum, (e) Ru 3p spectrum, (f) Co 2p spectrum in four materials.; Figure S6: Raman spectroscopy of the electrode material; Figure S7: The CV curves of (a) Co(OH)_2_-Se, (b) Ru-Co(OH)_2_, (c) Ru-Co(OH)_2_-Se catalyst materials at different scan rates, (d) The current density change value and the scanning rate of the three catalyst materials.; Figure S8: Timing potential curve of Ru-Co(OH)_2_-Se for 50 h in 30 wt% KOH solution.; Table S1: The content of Co, Se, Ru elements in Ru-Co(OH)_2_-Se.; Table S2: pH value of 1 M KOH. Reference [52] is cited in Supplementary Materials.
