# Supplementary material for: Ru and Se Co-Doped Cobalt Hydroxide Electrocatalyst for Efficient Hydrogen Evolution Reactions
Source: Molecules. 2023 Jul 28;28(15):5736. doi: 10.3390/molecules28155736 (PMC10420253; doi:10.3390/molecules28155736)
Supplement: Supplementary file 1 [file molecules-28-05736-s001.zip › molecules-2491029-supplementary.pdf]

Supporting Information

Ru and Se Co-Doped Cobalt Hydroxide Electrocatalyst for Efficient Hydrogen Evolution Reactions

Weizhong Peng, Yuting Yuan, Chao Huang, Yulong Wu, Zhaohui Xiao and Guanghui Zhan

OER and HER mechanism

The steps of OER primitives under alkaline conditions are as follows [1]:

$$OH^- + * \rightarrow OH_{ads} + e^- \quad (1)$$

$$OH_{ads} + OH^- \rightarrow O_{ads} + H_2O + e^- \quad (2)$$

$$O_{ads} + OH^- \rightarrow OOH_{ads} + e^- \quad (3)$$

$$OOH_{ads} + OH^- \rightarrow O_{2ads} + H_2O + e^- \quad (4)$$

$$O_{2ads} \rightarrow O_2 + * \quad (5)$$

$$\text{Overall: } 4OH^- \rightarrow 2H_2O + O_2 + 4e^-$$

The steps of OER primitives under acidic conditions are as follows:

$$H_2O + * \rightarrow OH_{ads} + H^+ + e^- \quad (1)$$

$$OH_{ads} \rightarrow O_{ads} + H^+ + e^- \quad (2)$$

$$O_{ads} + H_2O \rightarrow OOH_{ads} + H^+ + e^- \quad (3)$$

$$OOH_{ads} \rightarrow O_{2ads} + H^+ + e^- \quad (4)$$

$$O_{2ads} \rightarrow O_2 + * \quad (5)$$

$$\text{Overall: } 2H_2O \rightarrow 4H^+ + O_2 + 4e^-$$

where the * represents the active sites on the catalyst surface, and the "ads" represents adsorbed state of intermediates (OH_{ads}, O_{ads}, OOH_{ads}, and O_{2ads}).

The steps of HER primitives under alkaline conditions are as follows:

$$H_2O + e^- \rightarrow OH^- + H_{ads} \text{ (Volmer) } (1)$$

$$H_{ads} + H_2O + e^- \rightarrow OH^- + H_2 \text{ (Heyrovsky) } (2)$$

$$\text{or } 2H_{ads} \rightarrow H_2 \text{ (Tafel) } (3)$$

The steps of HER primitives under acidic conditions are as follows:

$$H^+ + e^- + * \rightarrow H_{ads} \text{ (Volmer) } (1)$$

$$H_{ads} + H^+ + e^- \rightarrow H_2 \text{ (Heyrovsky) } (2)$$

$$\text{or } 2H_{ads} \rightarrow H_2 \text{ (Tafel) } (3)$$

where the * represents the active sites on the catalyst surface, and the "ads" represents adsorbed state of intermediates (H_{ads}).

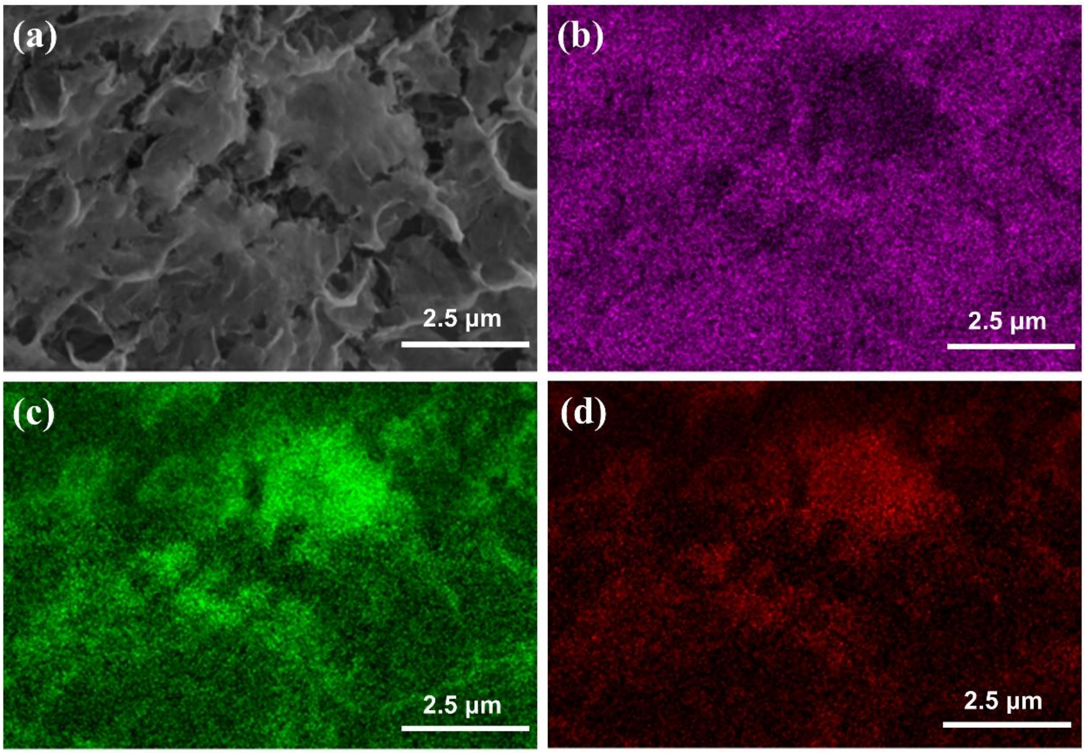

Figure S1. (**a**) SEM image of Ru-Co(OH)$_2$-Se. EDS elemental mapping images of (**b**) Co, (**c**) Se, (**d**) Ru.

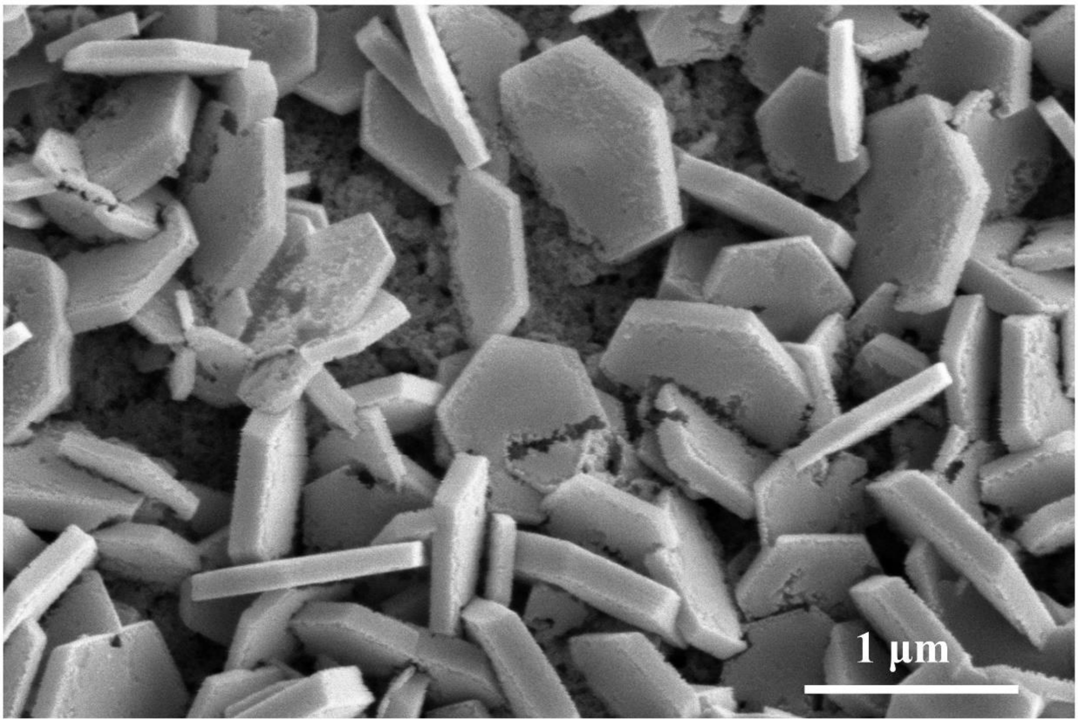

Figure S2. SEM image of post-HER Ru-Co(OH)$_2$-Se.

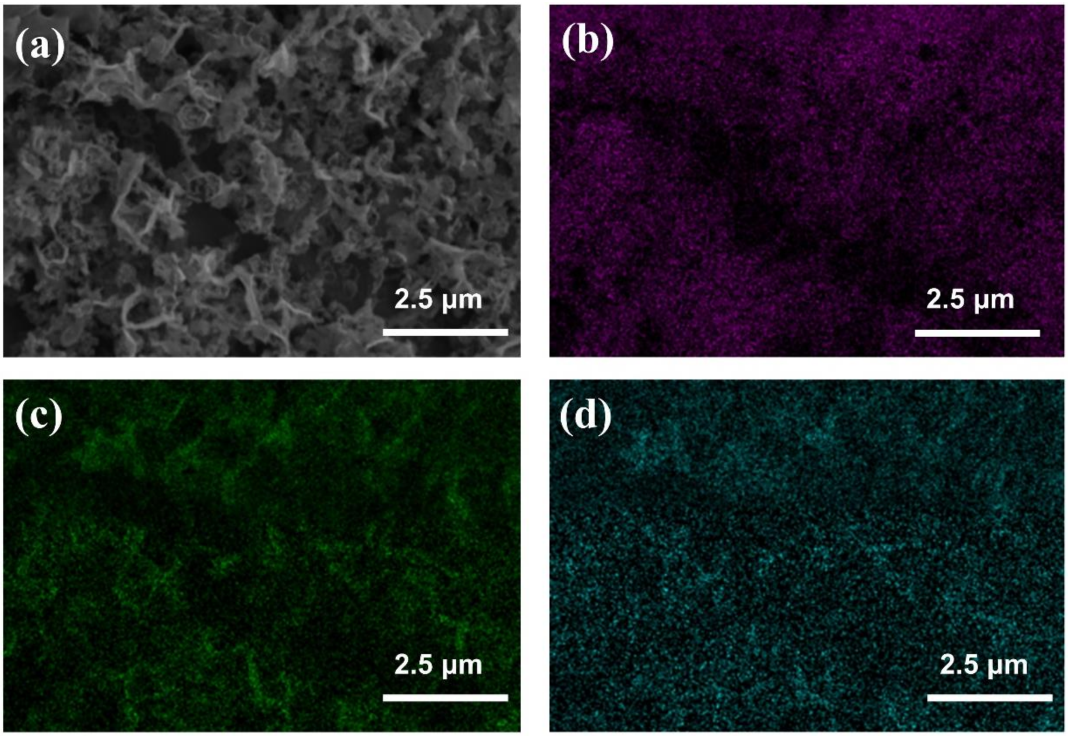

Figure S3. (**a**) SEM image of post-HER Ru-Co(OH)$_2$-Se. EDS elemental mapping images of (**b**)Co, (**c**)Se, (**d**)Ru.

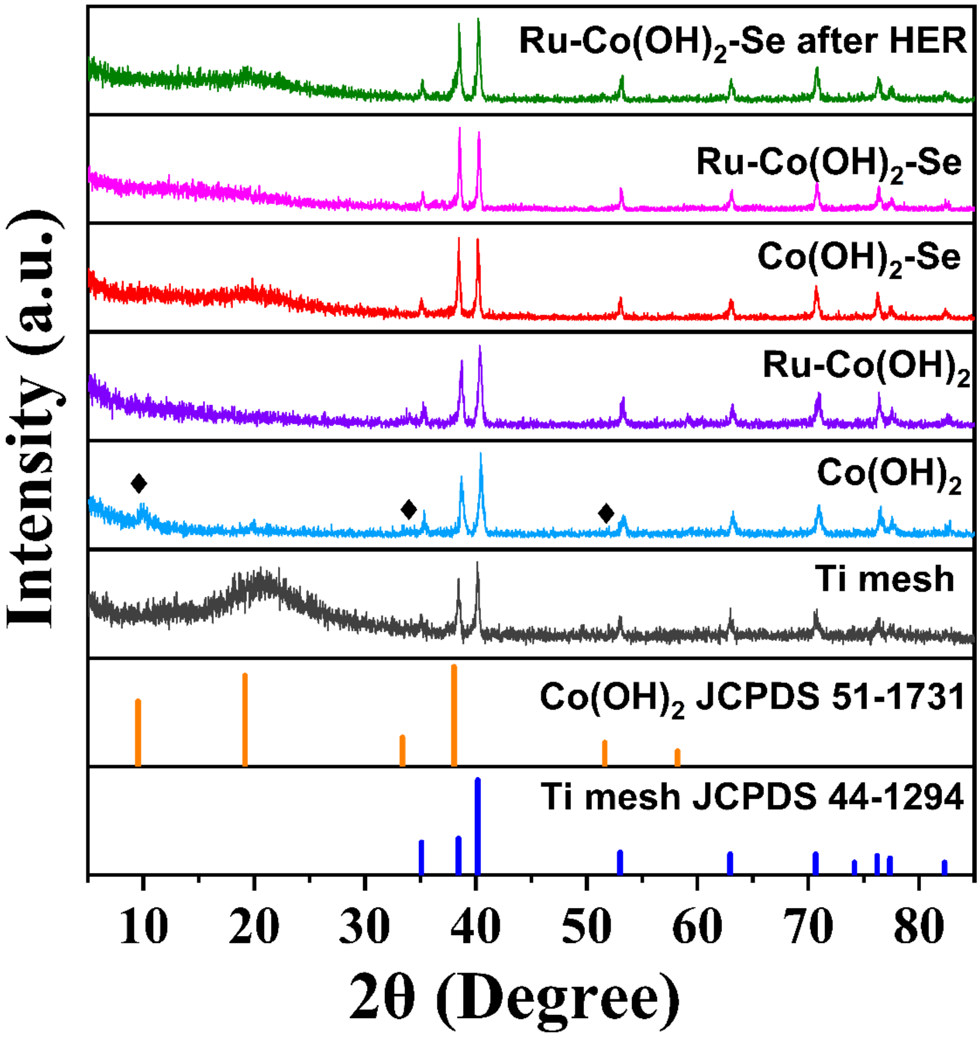

Figure S4. X-ray diffraction (XRD) patterns of each electrode material.

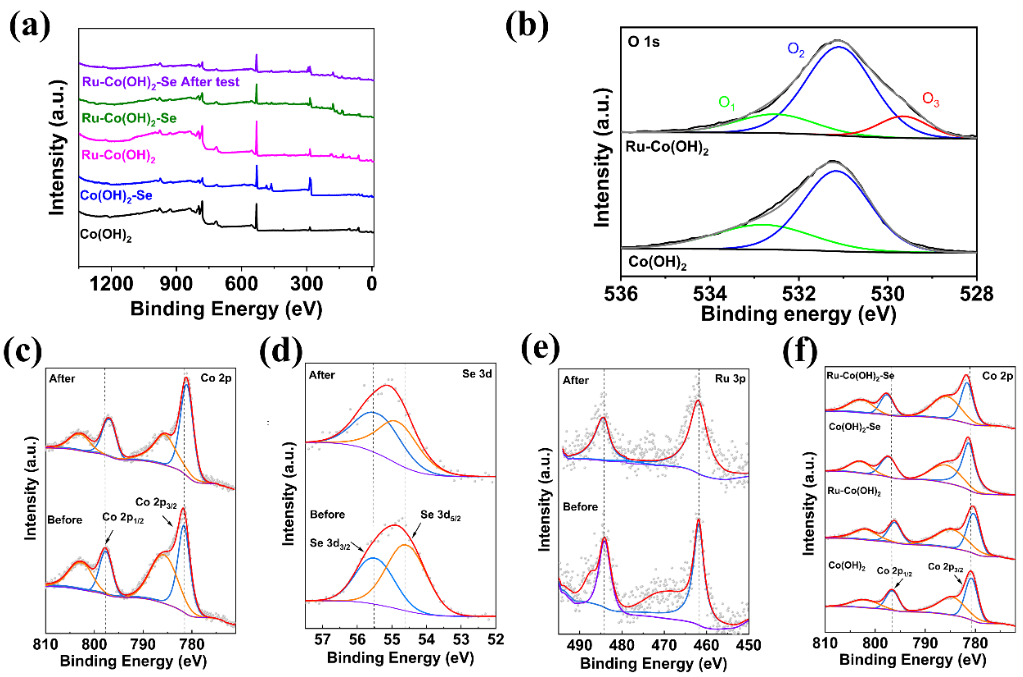

Figure S5. (**a**) XPS spectra of the electrode material, (**b**) O 1s spectrum, (**c**) Co 2p spectrum, (**d**) Se 3d spectrum, (**e**) Ru 3p spectrum, (**f**) Co 2p spectrum in four materials.

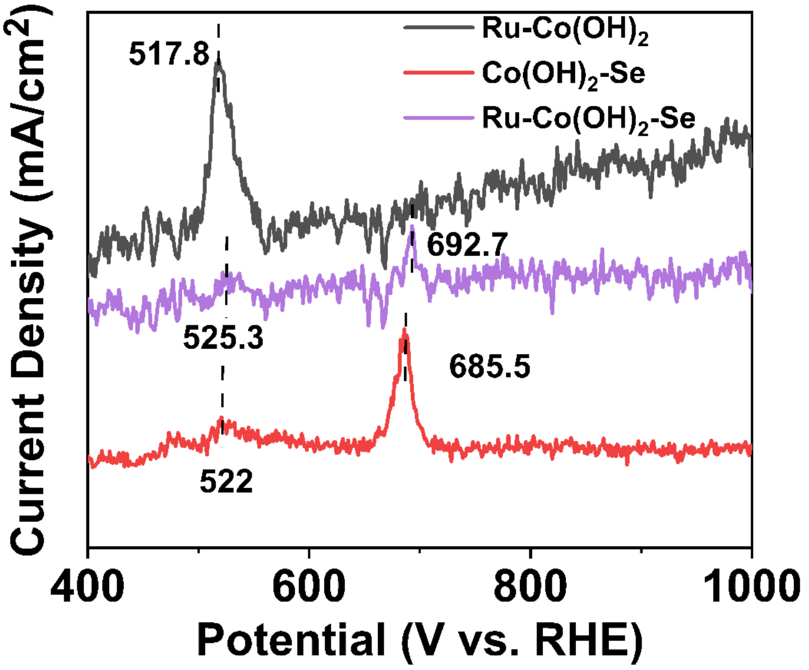

Figure S6. Raman spectroscopy of the electrode material.

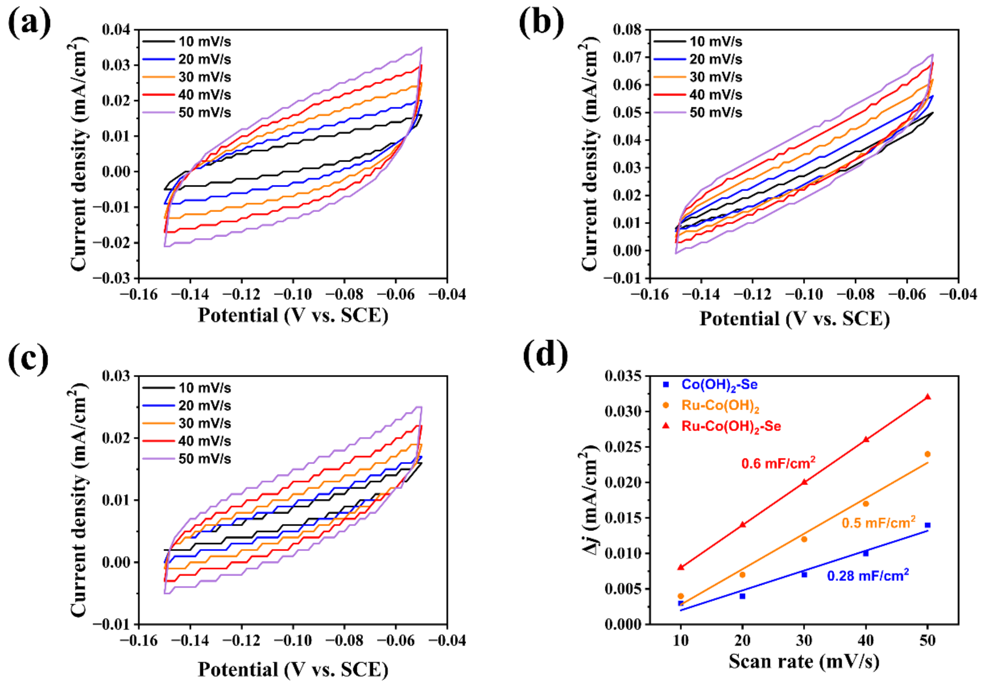

Figure S7. The CV curves of (**a**) Ru-Co(OH)$_2$-Se, (**b**) Ru-Co(OH)$_2$, (**c**) Co(OH)$_2$-Se catalyst materials at different scan rates.(**d**) The current density change value and the scanning rate of the three catalyst materials.

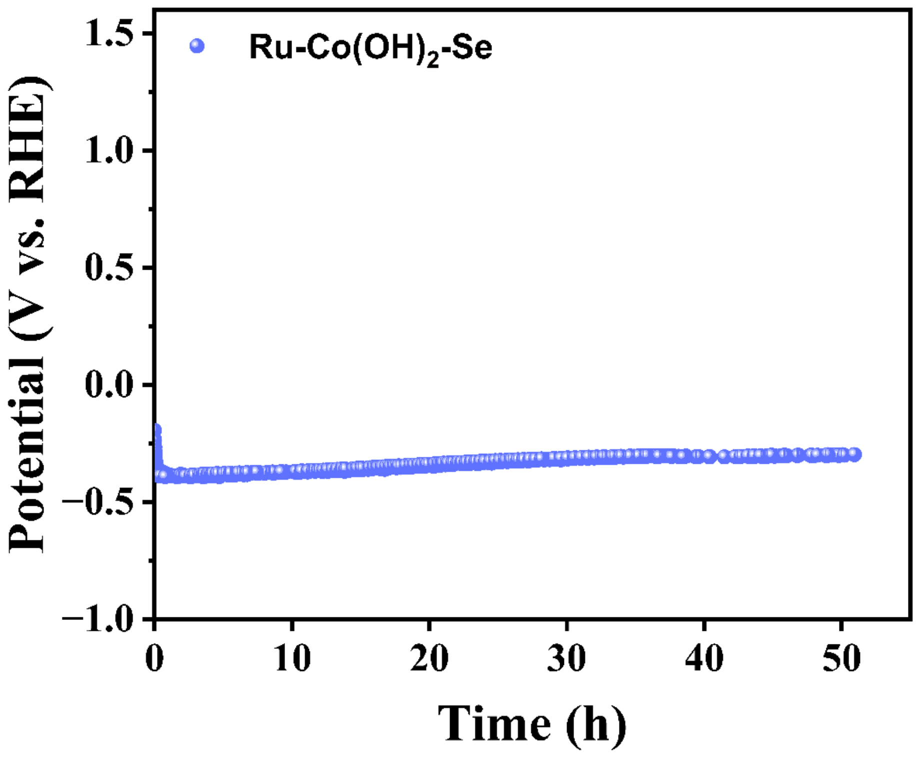

Figure S8. Timing potential curve of Ru-Co(OH)$_2$-Se for 50 hours in 30 wt% KOH solution.

Table S1. The content of Co, Se, Ru elements in Ru-Co(OH)$_2$-Se.

**Element**	**Wt %**	**σ**	**Mol %**
**Co**	64.7	0.1	0.27
**Se**	27.8	0.1	0.12
**Ru**	7.6	0.1	0.03

Table S2. pH value of 1M KOH.

1M KOH	Measured Value	Theoretical Value
**pH value**	13.77	14

1. Li, Z.; Li, B.; Yu, M.; Yu, C.; Shen, P. Amorphous metallic ultrathin nanostructures: A latent ultra-high-density atomic-level catalyst for electrochemical energy conversion. *Int. J. Hydrogen. Energy* **2022**, doi:10.1016/j.ijhydene.2022.06.049.
